# Evidence-Informed Deliberative Processes for HTA Around the Globe: Exploring the Next Frontiers of HTA and Best Practices Comment on "Use of Evidence-informed Deliberative Processes by Health Technology Assessment Agencies Around the Globe"

**DOI:** 10.34172/ijhpm.2020.145

**Published:** 2020-08-05

**Authors:** Unni Gopinathan, Trygve Ottersen, Pascale-Renée Cyr, Kalipso Chalkidou

**Affiliations:** ^1^Division for Health Services, Norwegian Institute of Public Health, Oslo, Norway.; ^2^Department of Community Medicine and Global Health, Institute of Health and Society, University of Oslo, Oslo, Norway.; ^3^Global Health Development Group, Imperial College London School of Public Health, London, UK.; ^4^Center for Global Development Europe, London, UK.

**Keywords:** Health Technology Assessment, Health Policy, Deliberative Processes, Decision-Making, Priority Setting

## Abstract

This comment reflects on an article by Oortwijn, Jansen, and Baltussen about the use and features of ‘evidence-informed deliberative processes’ (EDPs) among health technology assessment (HTA) agencies around the world and the need for more guidance. First, we highlight procedural aspects that are relevant across key steps of EDP, focusing on conflict of interest, the different roles of stakeholders throughout a HTA and public justification of decisions. Second, we discuss new knowledge and models needed to maximize the value of deliberative processes at the expanding frontiers of HTA, paying special attention to when HTA is applied in primary care, employed for public health interventions, and is produced through international collaboration.

## Introduction

Healthcare needs exceed available resources in every country.^1^ Setting efficient and equitable health priorities require both appropriate substantive criteria and fair process.^2^ To achieve the latter, scholars have argued the need for deliberative processes that satisfy key qualities, including transparency, broad involvement of stakeholders, consideration of outcomes valued by stakeholders, and mechanisms for appeal, revision and enforcement.^3,4^ National institutions that support evidence-informed priority setting, such as the United Kingdom’s National Institute of Health and Care Excellence (NICE), have to some extent institutionalized deliberative processes.^5^ Deliberative processes, guided by the Accountability for Reasonableness framework, have also been tested as part of priority setting at district levels in low- and middle-income countries.^6^

In their recent article, Oortwijn, Jansen and Baltussen (henceforth Oortwijn et al) present findings from a survey that asked members of the International Network for Agencies for Health Technology Assessment (INAHTA) about their use of deliberative processes.^7^ Oortwijn et al apply the term “evidence-informed deliberative processes” (EDPs) to describe how “HTA [health technology assessment] agencies should ideally organize their processes to achieve legitimate decision-making.”^7^ A general critique that can be leveled against the use of “EDP” to describe these processes is that it promotes the perception that it represents a “new” approach to explicitly addressing the issue of legitimacy, when it in fact involves qualities that resemble deliberative processes that have been set up in the context of healthcare priority setting at least for several decades.^4,5^

Oortwijn et al have previously developed a guide describing key steps of EDP: setting up an appraisal committee, defining decision criteria that reflect shared values and establishing a process for identifying and selecting health technologies for HTA, assessing and appraising a specific HTA, and finally communication and the opportunity for appeal.^8^ These steps form the basis of their survey of INAHTA members. Here, they investigate the extent to which INAHTA member agencies use the different steps of an EDP and the extent to which these agencies are in need of guidance for implementing these steps.

We provide two sets of reflections. First, we highlight key procedural aspects, tied to key steps addressed by the survey but that are relevant across these steps, where more guidance is needed. Second, we discuss new knowledge and models needed to maximize the value at the expanding frontiers of HTA, focusing on when HTA is applied in primary care, employed for public health interventions, and produced through international collaboration.

## Key Procedural Aspects in the Deliberative Process Where Guidance Is Needed

Through their survey, Oortwijn et al drew attention to a wide range of procedural aspects that define a deliberative process for HTA. Oortwijn et al tie these aspects to specific steps outlined in their model for EDP. In the following, we highlight three aspects relevant across the key steps of EDP, where efforts to define ‘best practices’ can generate value.

First, with respect to the first step of establishing a committee and stakeholder panel for appraising the health technology in question, between 38%-46% of the respondents requested guidance for addressing composition, terms, and selection, roles and responsibilities, and approaches. Clarity about these features is imperative to securing scientific independence and protecting a committee from undue influence of financial, institutional and intellectual interests.^9^ This is also important for clarifying the relative differences in power and potential influence of the stakeholders involved, so that HTA organizations can take this into account to uphold a fair process.

While Oortwijn and colleagues’ questionnaire raise the importance of stating conflict of interest, it did not explicitly ask HTA organizations about the policy they have in place for systematically identifying financial and non-financial interests held by stakeholders. Recent experiences among HTA organizations suggest such policies and good practice are crucial for maintaining integrity of the HTA process. For example, in France, reported conflicts of interest among members responsible for guidance development in the French national health agency (Haute Autorité de Santé) led to review and withdrawal of several guidelines.^10^ Moreover, direct industry influence commonly receive most attention; yet a recent study identified that funding by manufacturers of technologies under appraisal is highly prevalent among patient organisations contributing to HTA in England.^11^ While deliberation is considered integral to sound HTA, deliberation without clear policies for addressing conflict of interest risks doing more harm than good, for example via capture of the process by stakeholders with vested interests. A more detailed assessment of how the interests of different stakeholders are identified, made transparent, and managed when committees and stakeholder panels are formed would be useful for identifying best practice.

Second, the survey findings indicated a demand for more guidance about roles and responsibilities when different stakeholders are involved, especially with assessment and appraisal. The need for guidance on this matter point to several things. First, while stakeholder involvement is a key factor for promoting a fair process throughout scoping, assessment and appraisal, it is not necessarily so that the same type or number of stakeholders should cover these steps. For example, it is relevant that organizations representing the disease in question is involved to give input on what the key outcomes are, while a patient organizations representing diseases more broadly might be relevant to involve at a later state reflecting the need to evaluate priorities across diseases. Second, reflecting pivotal questions raised in a background paper for the 2020 HTAi Global Policy Forum, is whether deliberative processes afford opportunities to promote a wide range of values, whether participation allow for promoting competing interpretations of the need for the health intervention in question, and whether perspectives are integrated in such way that promotes learning among all the involved stakeholders.^12^

Third, over 90% indicated that HTA agencies make public decisions or make public to some extent the decisions and underlying reasons. Given the crucial role that publicity about the grounds for decisions play for a fair process, more careful scrutiny of how publicity is practiced is needed. Of particular importance is whether the agencies transparently report their data, models, social value judgements, and if the information is made available in a language accessible to the public.^6,13,14^ Moreover, to promote legitimacy, transparency is needed not only after decisions have been made but also throughout the HTA process.

There are limitations to using the survey findings to distill generalizable lessons. When surveying and comparing country experiences to assess the value of HTAs for health systems decisions, awareness of (1) the different nature of HTA organizations and (2) the extent to which HTAs and deliberative processes is put to use for the wide range of health systems decisions is important. The first speaks to the fact that HTA organizations across different countries have very different functions. While in the United Kingdom the decision-making processes and committees for reimbursements decisions informed by HTA rests within NICE, in other countries HTA organizations serve an advisory role responsible for evidence generation without decision-making authority nor influence over the design of the deliberative process.^15^ Moreover, given the wide range of experiences across differing contexts, it can be a better aim to identify a ‘package’ of best practices since sound practice in one setting might not be easily transferable to another.

The second point speaks to the fact that a range of health systems decisions function without the use of HTA or similar evidence-informed assessments nor a deliberative process. This includes, for example, health financing choices related to revenue generation and pooling, where the need for explicit and deliberative processes have received less scrutiny than for purchasing. Moreover, implicit priority setting frequently occur when resources are allocated without being subject to deliberations where evidence of benefits and harms, assumptions, trade-offs and social values are made explicit. The latter is for example true for resources allocated to countries by the Global Fund, which is yet to systematically make use of HTAs when determining which interventions to support.^16,17^

We agree with the authors that a response rate covering a little more than half (54%) of INAHTA members might be hiding two types of gaps. First, the lack of response risks hiding weaknesses among other agencies, which would suggest that a greater number of agencies than those who responded are in need of guidance about various aspects of designing a sound deliberative process. Second, experiences beyond those surveyed should be investigated in order to obtain a global view. From a total of 50 INAHTA member agencies in 2018, Oortwijn et al received a complete response from 25 agencies, and the majority of these (15) were European HTA agencies. In spite of the recent growth of HTA agencies in Asia and Latin America, only seven agencies from these two regions left a response. In comparison, an other member organization in Asia, HTAsialink, have 24 agencies from 15 countries: Australia, Bhutan, China, Indonesia, Japan, Kazakhstan, Malaysia, Mongolia, New Zealand, Philippines, Korea, Singapore, Taiwan, Thailand, and Vietnam. It is crucial that the perspectives of these and other agencies on good practice with respect to deliberative processes are considered.

## Opportunities Going Forward: Exploring the Next Frontiers of HTA

Universal health coverage is a widely promoted policy goal in the sustainable development agenda. In response, countries at different income levels are increasingly setting up institutions to facilitate explicit priority setting processes informed by HTAs when allocating scarce health resources.^19-22^ To facilitate sharing of country experiences and to identify factors that can promote the use of HTA, detailed national and regional assessments of HTA institutionalization haven taken place.^23,24^ Moreover, capacity among policy-makers to make use of HTA and deliberate with other stakeholders is sought strengthened in different settings.^23,25^ Oortwijn et al promote a view that key features of a deliberative process should be present at every step of prioritizing health interventions and at every level where health interventions are prioritized. This position is shared by many,^12,18^ and promising experiences suggest that the frontiers of HTA is being expanded.

First, the most frequent use of HTA is to inform public reimbursement decisions for health technologies used in specialist healthcare. However, countries are increasingly considering how priority-setting can be done across different levels of care, and how HTA can be applied to services delivered at the municipal level, especially primary care services. For example, in 2018, a national committee mandated by the Norwegian government proposed the use of criteria and processes similar to specialist healthcare for priority setting of health services, including preventive services, at the municipal level.^26^

Moreover, a few countries are now experimenting with approaches at the level of local government that facilitate involvement of end users at the point of topic selection. For example, in Norway, a pilot has been implemented among 11 municipalities to motivate the use of evidence to inform decisions about health and social services, and public health.^27^ A unique feature of this pilot was to generate a demand for research evidence among decision-makers in the municipalities. Frontline providers and local policy-makers were motivated to suggest interventions, ideally ones they are considering for implementation, for assessment of evidence by the Norwegian Institute of Public Health. Similarly, the UK’s National Institute of Health Research is developing a programme — the Public Health Intervention Responsive Studies Teams — to support evidence-informed delivery and implementation of public health interventions.^28^ A key feature of these initiatives is the emphasis on joint ownership, whereupon researchers and local policy-makers jointly prioritize topics and consider the value of evaluations.

Second, the use of HTAs for public health interventions is receiving growing attention,^29,30^ and presents its own set of challenges and opportunities with respect to deliberative processes for HTA. For example, stakeholder involvement can be particularly demanding for public health interventions. These interventions typically demand involvement of different sectors, affect interests of different sectors, and require explicit consideration of outcomes beyond health.^31^ Moreover, in many settings, the responsibility for delivering public health interventions has been decentralized to local government (eg, municipal level), where the use of HTAs and evidence-informed guidance during deliberative processes is immature and evolving.^32^ Overall, for public health decisions, the scope of actors involved with EDPs is likely to be even wider than for clinical care.

Finally, increasing international collaboration, particularly shared functions and joint production to promote efficient use of HTAs, is expected to push the frontiers of HTA, while posing new challenges and opportunities for deliberation. For example, deliberation at the stage of horizon scanning has been less explored than deliberation during the process of conducting an HTA, but several major international HTA collaborations have piloted joint processes and deliberation as part of horizon scanning. In the European setting, the Beneluxa collaboration, involving Belgium, the Netherlands, Luxembourg, Austria and Ireland, aims to promote joint horizon scanning for emerging drugs and health technologies.^33^ Moreover, the collaboration EUnetHTA is piloting joint topic identification, selection and prioritization as part of testing joint horizon scanning functions.^34^ The role of horizon scanning was also the theme of the 2019 HTAi policy forum in Asia, which underscored the need for a transparent horizon scanning process that fosters early dialogue among HTA agencies, providers and industry about technologies in a clinical pathway, and the need for a shared Asian horizon scanning network.^35^

## Conclusion

Ooortwijn et al have provided an early baseline assessment of the experience HTA organizations worldwide have with different steps of the deliberative process for HTAs. Using their work as point of departure, we have highlighted critical procedural aspects — managing conflict of interest, clarifying the different roles of stakeholders at different steps, and public justification of decisions — where efforts to define best practices can generate value. Defining such practices should consider the different roles HTA agencies take on during decision-making processes in health systems, and be informed by in-depth comparative work of experiences with the next frontiers of HTA, including when HTA is applied in primary care, employed for public health interventions, and is produced through international collaboration.

## Acknowledgements

We thank the two peer-reviewers for providing comments that helped improve the quality of the manuscript.

## Ethical issues

Not applicable.

## Competing interests

UG and TO work for the Norwegian Institute of Public Health, which is an INAHTA member.

## Authors’ contributions

All authors contributed to drafting the manuscript and critically revising it for intellectual content. All authors read and approved the final manuscript.

## Authors’ affiliations

^1^ Division for Health Services, Norwegian Institute of Public Health, Oslo, Norway. ^2^ Department of Community Medicine and Global Health, Institute of Health and Society, University of Oslo, Oslo, Norway.^3^ Global Health Development Group, Imperial College London School of Public Health, London, UK. ^4^ Center for Global Development Europe, London, UK.
